# Should Emergent Pericardiocentesis Be Added to Pediatric ICU Training?

**DOI:** 10.1155/cric/8965488

**Published:** 2026-03-04

**Authors:** Lanah Almatroud, Waseem Ostwani

**Affiliations:** ^1^ Michigan State University College of Human Medicine, East Lansing, Michigan, USA, msu.edu; ^2^ Division of Critical Care Medicine, Department of Pediatrics, University of Toledo College of Medicine & Life Sciences, Toledo, Ohio, USA

**Keywords:** child care, critical care, medical education

## Abstract

Purulent pericarditis can be rapidly fatal if not diagnosed and treated promptly. We present a case of purulent pericarditis that progressed to cardiac tamponade, necessitating emergent bedside pericardiocentesis. Currently, pericardiocentesis is not a core component of the Graduate Medical Education in Pediatric Critical Care Medicine (PCCM) in the United States. We recommend incorporating pericardiocentesis training into the PCCM fellowship curriculum.

## 1. Introduction

We present a case where subtle symptoms of bacterial pericarditis rapidly progressed to cardiac tamponade with obstructive shock, necessitating urgent drainage via pericardiocentesis. Purulent pericarditis (PP) can be rapidly fatal if not promptly diagnosed and treated [1]. Typically characterized by fever, tachycardia, cough, and occasionally chest pain, PP can lead to complications such as cardiac tamponade [2]. Pericardiocentesis is considered the preferred diagnostic and therapeutic intervention for PP, especially in the presence of tamponade [3].

## 2. Case Presentation

A previously healthy 6‐year‐old male presented to the emergency department (ED) with right‐sided chest pain, shortness of breath, and abdominal pain that awoke him from sleep a few hours before arrival. On examination, his heart rate (HR) was 115/min, respiratory rate (RR) was 35/min, blood pressure (BP) was 96/54 mmHg, temperature was 97.9°F, and oxygen saturation was 99% on room air. His white blood cell (WBC) count was mildly elevated at 14.1 × 10^9^/L (normal range: 4.5–13.5 × 10^9^/L).

The patient was alert and interactive but exhibited shallow respirations, marked abdominal distension with normal bowel sounds, and no peritoneal signs. Chest radiography showed a normal cardiac silhouette and lung fields. An electrocardiogram (ECG) was also normal. Abdominal radiography revealed stool throughout the colon. A diagnosis of constipation was made, and the patient was treated with a dose of polyethylene glycol and discharged home.

The patient returned to the ED 13 h later with three episodes of nonbloody, nonbilious vomiting and worsened abdominal pain. At this visit, he was tachycardic and tachypneic, with a HR of 150/min, RR of 34/min, BP of 118/60 mmHg, temperature of 99.1°F, and oxygen saturation of 100% on room air. Examination revealed grunting respirations with nasal flaring, diffuse abdominal pain without guarding or rebound tenderness, and negative Murphy′s and Rovsing′s signs.

Upon return to the ED, laboratory results revealed significant findings: WBC of 16.6 × 10^9^/L, C‐reactive protein (CRP) of 5.7 mg/dL (normal range: < 0.744 mg/dL), erythrocyte sedimentation rate (ESR) of 31 mm/h (normal range: 0–10 mm/h), procalcitonin of 10.42 ng/mL (normal range: < 0.05 ng/mL), fibrinogen of 536 mg/dL (normal range: 190–480 mg/dL), activated partial thromboplastin time (aPTT) of 33 s (normal range: 26–37 s), prothrombin time (PT) of 26.7 s (normal range: 9.8–13.2 s), and international normalized ratio (INR) of 2.3 (normal range: 0.8–1.1). Serum electrolytes, amylase, lipase, hemoglobin, urinalysis, and lactate levels were within normal ranges.

The patient received a normal saline fluid bolus and intravenous doses of ketorolac and acetaminophen, leading to improvement in pain and distress. He was then admitted to the pediatric floor for further management. Abdominal MRI revealed fluid collection around the appendix, gallbladder, and in the lower abdomen and pelvis but no gallstones. Abdominal ultrasound showed a thickened wall and an ill‐defined appendix, with no other abnormalities. Empirical antibiotics (piperacillin and tazobactam) were started, along with intravenous acetaminophen, ondansetron, famotidine, and vitamin K.

Four hours later, the patient developed a fever (101.1°F), tachycardia (153/min), tachypnea (30 breaths/min), and with a BP of 94/40 mmHg. He exhibited signs of distress, including increased work of breathing, nasal flaring, grunting, and anterior left‐sided chest pain. His HR rose to 170/min. Bedside ECG showed diffuse ST elevation, and he had muffled heart sounds with right upper quadrant tenderness and guarding. The patient received an additional normal saline bolus (10 cc/kg) and was urgently transferred to the pediatric intensive care unit (PICU).

In the PICU, signs of obstructive cardiogenic shock were evident, with bedside echocardiogram revealing pericardial effusion and right atrial collapse. Cardiac tamponade was confirmed by a pediatric cardiologist, and urgent transport to the nearest pediatric cardiac intensive care unit (CICU) was arranged. Despite an additional 10 cc/kg normal saline bolus, the patient remained hemodynamically unstable with systolic BP in the 60s–70s, HR in the 170s, and capillary refill > 5 s. He became extremely lethargic, and pericardiocentesis was initiated emergently under strict sterile technique with real‐time ultrasound guidance. The patient received 15 mg of intravenous ketamine for procedural sedation and analgesia. A 21‐gauge needle from a Micropuncture Introducer Set was initially inserted in an out‐of‐plane approach using ultrasound guidance to ensure precise placement into the pericardium. Once the needle entered the pericardial space, a guidewire was advanced through the needle, which was then removed. The introducer set was inserted over the guidewire, followed by the removal of the inner dilator. The guidewire for the pericardial drainage catheter (8.5 Fr, 15 cm) was then introduced, and following dilation, a pigtail catheter was advanced over the guidewire. Approximately 300 mL of purulent fluid was aspirated, with postprocedural echocardiographic monitoring showing a reduction in effusion volume and improvement in hemodynamics (HR dropped to the 120s, systolic BP increased to the 90s).

The patient showed clinical improvement, with responsiveness returning. This case highlights the importance of pericardiocentesis as a core procedural competency in pediatric critical care, demonstrating both technical proficiency and its impact on patient outcomes.

Following stabilization, the patient was transferred to the CICU and underwent a subxiphoid pericardial window procedure 2 days later. He was discharged on intravenous ceftriaxone for a 30‐day course and followed up with pediatric cardiology 1 month after discharge, showing good recovery and a normal echocardiogram.

## 3. Discussion

We present a case with subtle symptoms of bacterial pericarditis that advanced quickly to cardiac tamponade with obstructive shock requiring urgent drainage via a pericardiocentesis catheter. Several studies highlight the critical role of urgent and emergent pericardiocentesis in managing PP and tamponade, particularly when rapid transfer to a larger institution is not feasible. Simonÿ et al. reported a case where bedside pericardiocentesis was lifesaving and facilitated the patient′s transfer to a larger hospital for further care [4]. Similarly, Lutmer et al. described two pediatric cases of PP managed with emergent bedside pericardiocentesis, leading to rapid hemodynamic improvement and resolution of refractory shock [5]. In contrast, Maffione et al. highlighted cases where bedside pericardiocentesis was not feasible, necessitating transfer to a Cardiac Surgery Unit at another hospital. The transfer was only possible due to the patient′s stable hemodynamic condition [6].

Currently, pericardiocentesis is not a core requirement in the Graduate Medical Education curriculum for Pediatric Critical Care Medicine (PCCM) in the United States. Fellows in PCCM programs are expected to perform core procedures, including peripheral and central catheterization, endotracheal intubation, thoracostomy tube placement, resuscitation, and procedural sedation [7]. However, many PICUs are located in urban and suburban areas that may not have direct access to specialized cardiac care. This gap poses a significant risk, as the inability to perform a lifesaving procedure like pericardiocentesis can be fatal if immediate transfer to a larger facility is not possible [8].

Given that pericardiocentesis is a relatively infrequent but complex procedure, high proficiency retention is crucial. Simulation‐based training has been shown to improve retention rates for similar procedures [9]. We recognize that pericardiocentesis is an infrequent but potentially lifesaving procedure, and therefore, opportunities for clinical exposure during training may be limited. There is growing support for incorporating pericardiocentesis training into fellowship curricula, particularly given the procedure′s rarity and high stakes. While there is limited existing literature specifically addressing the integration of pericardiocentesis into pediatric critical care fellowship education, there is increasing evidence and precedent for simulation‐based training for this and analogous procedures like thoracostomy tube placement or central line insertion. Studies show that low‐cost, high‐fidelity models can effectively simulate both ultrasound‐guided and landmark‐based pericardiocentesis, allowing repeated practice and improving trainee competence [10–16]. For instance, a 3D‐printed cardiac model embedded in an ultrasound‐compatible gelatin block has been shown to simulate the procedure realistically, with the majority of participants successfully placing a pericardial drain [17]. Moreover, there are models for both ultrasound and landmark‐guided techniques, adaptable for resource‐limited settings [18]. Simulation training has also been shown to increase both confidence and procedural success, though periodic retraining is necessary to maintain skills [12, 15]. Based on these findings, we strongly advocate for the inclusion of focused pericardiocentesis training in pediatric critical care fellowships, utilizing both didactic and simulation‐based training to ensure comprehensive skill development in fellows. This approach is particularly vital in pediatric ICUs where rapid intervention is essential and transferring patients may not always be feasible.

## 4. Conclusion

This case illustrates the rapid progression from PP to cardiac tamponade, emphasizing the critical need for early diagnosis and timely intervention. The patient′s condition escalated from initial symptoms to obstructive cardiogenic shock, necessitating urgent pericardiocentesis, which was crucial for stabilization and recovery.

The findings emphasize the importance of including pericardiocentesis training in PCCM fellowship programs. Currently, this procedure is not a core component of US fellowships, potentially leaving a gap in essential skills. Integrating focused training and simulation‐based practice will better equip future intensivists to manage severe cases effectively, particularly when immediate transfer to specialized care is not feasible.

## Funding

No funding was received for this manuscript.

## Consent

Written informed consent to participate in this study was provided by the patient′s legal guardians.

## Conflicts of Interest

The authors declare no conflicts of interest.

## Data Availability

Data sharing is not applicable to this article as no datasets were generated or analyzed during the current study.
